# TSPO PET detects acute neuroinflammation but not diffuse chronically activated MHCII microglia in the rat

**DOI:** 10.1186/s13550-020-00699-x

**Published:** 2020-09-29

**Authors:** Nassir U. Al-Khishman, Qi Qi, Austyn D. Roseborough, Alexander Levit, Brian L. Allman, Udunna C. Anazodo, Matthew S. Fox, Shawn N. Whitehead, Jonathan D. Thiessen

**Affiliations:** 1 Department of Medical Biophysics, Schulich School of Medicine and Dentistry, Western University, London, ON Canada; 2 Department of Anatomy and Cell Biology, Schulich School of Medicine and Dentistry, Western University, London, ON Canada; 3 Department of Physics and Astronomy, Western University, London, ON Canada; 4 Research Centre for Studies in Aging, McGill University, Montreal, QC Canada; 5 Lawson Health Research Institute, B5-003a, 268 Grosvenor St, Stn. B, P.O. Box 5777, London, ON N6A 4V2 Canada

**Keywords:** TSPO, Major histocompatibility complex class II, White matter inflammation, Ischemic stroke, Alzheimer’s disease

## Abstract

**Background:**

Accurate and sensitive imaging biomarkers are required to study the progression of white matter (WM) inflammation in neurodegenerative diseases. Radioligands targeting the translocator protein (TSPO) are considered sensitive indicators of neuroinflammation, but it is not clear how well the expression of TSPO coincides with major histocompatibility complex class II (MHCII) molecules in WM. This study aimed to test the ability of TSPO to detect activated WM microglia that are immunohistochemically positive for MHCII in rat models of prodromal Alzheimer’s disease and acute subcortical stroke.

**Methods:**

Fischer 344 wild-type (n = 12) and TgAPP21 (n = 11) rats were imaged with [^18^F]FEPPA PET and MRI to investigate TSPO tracer uptake in the corpus callosum, a WM region known to have high levels of MHCII activated microglia in TgAPP21 rats. Wild-type rats subsequently received an endothelin-1 (ET1) subcortical stroke and were imaged at days 7 and 28 post-stroke before immunohistochemistry of TSPO, GFAP, iNOS, and the MHCII rat antigen, OX6.

**Results:**

[^18^F]FEPPA PET was not significantly affected by genotype in WM and only detected increases near the ET1 infarct (*P* = 0.033, infarct/cerebellum uptake ratio: baseline = 0.94 ± 0.16; day 7 = 2.10 ± 0.78; day 28 = 1.77 ± 0.35). Immunohistochemistry confirmed that only the infarct (TSPO cells/mm^2^: day 7 = 555 ± 181; day 28 = 307 ± 153) and WM that is proximal to the infarct had TSPO expression (TSPO cells/mm^2^: day 7 = 113 ± 93; day 28 = 5 ± 7). TSPO and iNOS were not able to detect the chronic WM microglial activation that was detected with MHCII in the contralateral corpus callosum (day 28 OX6% area: saline = 0.62 ± 0.38; stroke = 4.30 ± 2.83; *P* = .029).

**Conclusion:**

TSPO was only expressed in the stroke-induced insult and proximal tissue and therefore was unable to detect remote and non-insult-related chronically activated microglia overexpressing MHCII in WM. This suggests that research in neuroinflammation, particularly in the WM, would benefit from MHCII-sensitive radiotracers.

## Introduction

Diffuse inflammation of brain white matter (WM) has been clinically implicated in the pathology of neurodegenerative diseases including schizophrenia, traumatic brain injury, and Alzheimer’s Disease [1–3]. In the continuum from mild cognitive impairment to Alzheimer’s disease (AD), diffuse WM inflammation may explain why WM undergoes demyelination, tract disintegration, and atrophy while patients undergo cognitive decline [4–6]. To measure WM inflammation in living subjects, previous studies have used PET tracers that target the 18 kDa translocator protein (TSPO) [7, 8]. TSPO is overexpressed in astrocytes and microglia during their activation, but the subtypes of activated microglia that express TSPO remain a subject of research.

Microglia that express major histocompatibility complex class II (MHCII) molecules, which are involved in antigen presentation, are considered to be proinflammatory [9]. MHCII molecules are correlated to cognitive dysfunction throughout AD and diffusely overexpressed in the WM microglia of patients with early-onset AD [1, 10]. Previously, our group correlated diffuse MHCII activated microglia in WM with cognitive dysfunction in a prodromal transgenic rat model of AD that overexpresses human Swedish- and Indiana-mutated amyloid precursor protein (TgAPP21) to produce high levels of beta-amyloid without depositing amyloid plaques [11–13]. We further demonstrated the importance of diffuse MHCII activated microglia by detecting them in WM remote to an ischemic subcortical stroke in an endothelin-1 (ET1) rat model that also exhibits executive dysfunction [14, 15]. Overall, these observations motivate the need to detect MHCII activated microglia in vivo.

In this study, we used the TgAPP21 and ET1-induced subcortical stroke rat models to investigate whether TSPO can detect diffuse MHCII activated microglia in WM. To quantify TSPO in vivo, we used a second-generation TSPO PET tracer ([^18^F]FEPPA) and validated the cerebellum as a pseudoreference region [16]. TSPO-PET was validated using immunohistochemistry of TSPO, which was compared to the activated astrocyte marker GFAP and activated microglia markers iNOS and OX6 (MHCII) in ET1 rats.

## Materials and methods

### Animals and experimental design

Animal ethics and procedures of this study are in compliance with the Canadian Council for Animal Care and were approved by the Western University Animal Care Committee (Protocol 2014-016). Rats were housed under a 12-h/12-h light/dark cycle and received ad libitum access to food and water.

This study used male rats of the Fischer 344 strain aged 11–14 months. TgAPP21 (n = 11) and wild-type (n = 12) rats were used to investigate [^18^F]FEPPA uptake in WM of prodromal AD using PET. Randomization was accomplished using rat ids. Genotype was validated with PCR as previously described [13].

**Fig. 1:**
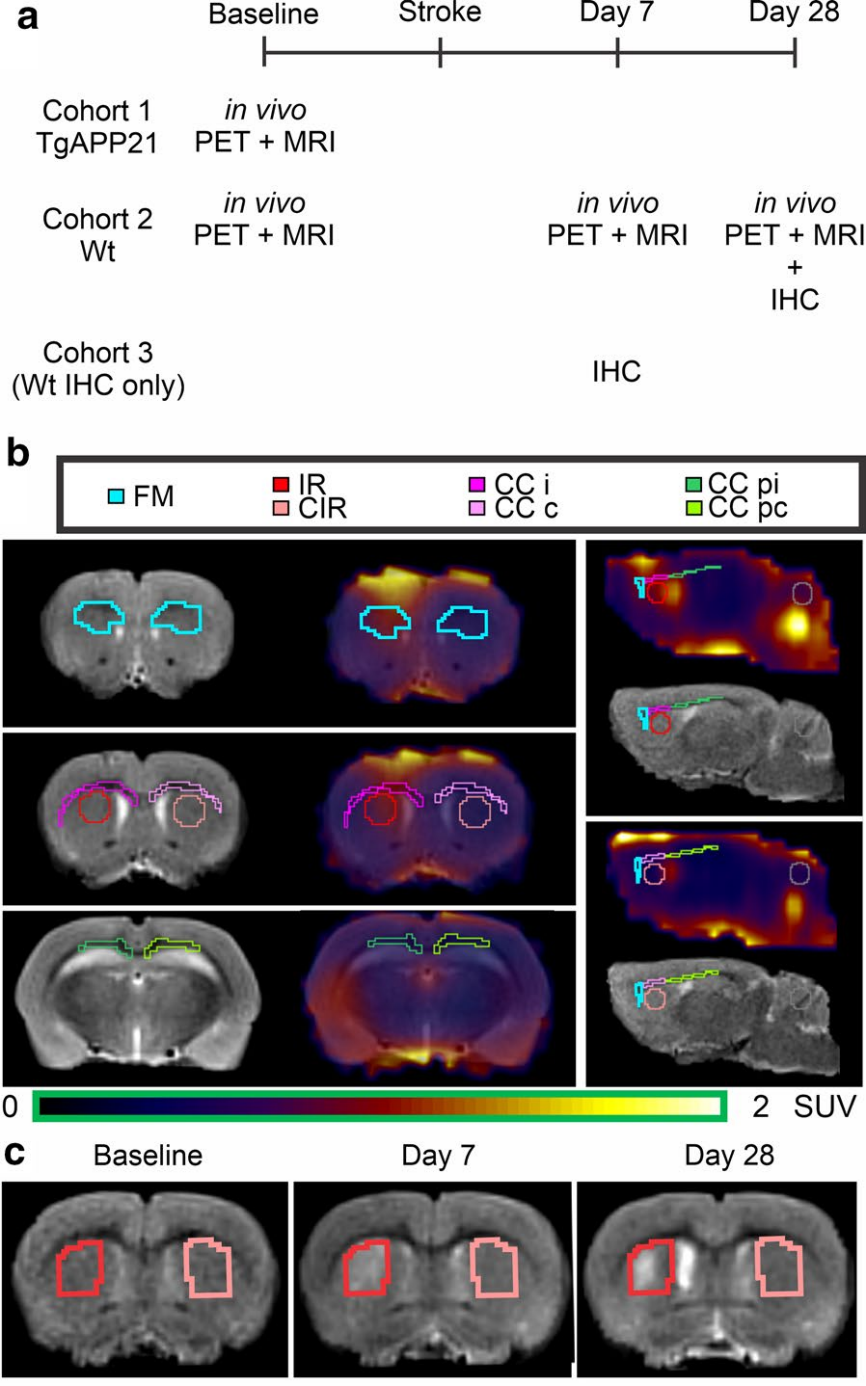
Analytical methods. **a** Experimental timeline. **b** Representative ROIs delineated on T_2_w MRI and applied to fused [^18^F]FEPPA PET in a control rat at day 7. **c** Infarct at baseline and post-stroke day 7 and 28 on T_2_w MRI. c, contralateral; CC, corpus callosum; CIR, contralateral to the infarct region, FM: Forceps Minor; i, ipsilateral; IR, infarct region p, posterior

To study ischemic subcortical stroke, the same wild-type rats that received baseline in vivo imaging later received a stereotactic injection of 60 pmol endothelin-1 (ET1) dissolved in 3 μL sterile saline (n = 5) or sterile saline alone for control (n = 6) in the right dorsal striatum as previously described [17]. Whether a rat received ET1 or only saline was randomized. Rats were imaged in vivo post-stroke at day 7 and day 28, at which point they were euthanized for immunohistochemistry (Fig. 1a). A saline rat was omitted at day 7 because of a tail vein issue and 2 saline rats had to be replaced for imaging on day 7 and day 28 because they died between initial imaging and surgery. Their baseline points were included in the figures and all analyses where applicable. For day 7 immunohistochemistry, additional rats were euthanized at day 7 (n = 5 ET1, n = 6 saline).

### In vivo imaging

Each in vivo imaging session consisted of a PET scan followed by MRI and CT. Before PET, anesthesia was administered at 2 L/min oxygen with isoflurane at 5% for induction then maintained at 2% until after CT. Respiratory rate, heart rate, and body temperature were monitored and maintained throughout.

### MRI

MRI was performed on a 3T MRI (Siemens Biograph mMR) with a dedicated quadrature rat brain coil (Cubresa, 44 mm inner diameter). 2D sagittal and axial T_2_-weighted fast spin echo (FSE) images were acquired spanning the brain with an in-plane field-of-view (FOV) = (70 × 70) mm^2^, matrix size = 320 × 320, in-plane resolution = (0.22 × 0.22) mm^2^ and slice thickness = 1 mm. Other relevant parameters for the FSE sequence include: repetition time (TR) = 2930 ms (sagittal acquisition) and 3900 ms (axial acquisition), effective echo time = 97 ms, refocusing flip angle = 150°, echo train length = 11, bandwidth = 225 Hz/pixel, and averages = 4.

### CT

For attenuation correction of the PET, full-body helical CT scans were acquired with a resolution of (0.20 × 0.20 × 1.25) mm^3^, tube current = 80 mA, tube voltage = 80 kVp, and rotation time = 1 s (GE Revolution CT).

### PET acquisition and processing

PET data were acquired dynamically over 90-min (Siemens Inveon). Thirty seconds after initiating the scan, 33.7 ± 5.6 MBq [^18^F]FEPPA was injected through a tail vein catheter. On day 28, rat tail arteries were cannulated for blood sampling (n = 4). A blood volume of 50 µL was sampled at 2, 8, 16, 64, and 90 min post-injection. Blood was centrifuged to extract plasma, which was then passed through omniphilic cartridges at 0%, 20%, and 40% acetonitrile to elute successively more lipophilic metabolites (Oasis HLB, Waters) [18]. Elutants, blood, and plasma were counted using a calibrated gamma detector.

PET data from 70–90 min post-injection were used for standardized uptake value (SUV) analysis and dynamic frames of 1 × 27 s, 21 × 3 s, 12 × 10 s, 5 × 30 s, 4 × 60 s, and 16 × 300 s for pharmacokinetic modeling, in which the radiotracer was not injected until after the first thirty seconds. The 70–90 min timeframe was chosen for static quantification because it was sufficiently long to provide a sufficient signal-to-noise and, given our acquisition, it was the latest timeframe possible, which is often the gold standard [19]. PET reconstruction consisted of 2 iterations of 3D ordered-subset expectation-maximization followed by 18 iterations of fast maximum a posteriori (OSEM3D/FMAP) with a co-registered CT-based attenuation correction map.

### PET analysis

Co-registration, region of interest (ROI) segmentation, and data extraction were performed using 3D Slicer 4.10 [20]. Images were zero-interpolated to an isotropic 0.22 mm voxel size and linearly co-registered to define the ROI in each rat (Fig. 1). ROI segmentation was based on the Paxinos-Watson atlas and included the T_2_w-hyperintense infarct region and contralateral to the infarct region for a positive control (5.39 ± 3.09 mm^3^), periaqueductal gray (4.09 ± 0.04 mm^3^), cerebellum (29.97 ± 4.50 mm^3^), frontal cortex (17.54 ± 3.95 mm^3^ per side), WM forceps minor (2.37 ± 0.65 mm^3^ per side), corpus callosum proximal to the infarct (5.03 ± 1.25 mm^3^ per side), and corpus callosum remote to the infarct (7.59 ± 1.85 mm^3^ per side). A 4 mm^3^ spherical ROI was placed in the center of the cardiac left ventricular cavity to derive input functions. Input functions were corrected using biexponential and Hill function population fits of whole blood-to-plasma ratio and unmetabolized [^18^F]FEPPA-to-all metabolites ratio (MATLAB 2019a). Corrected input functions and time–activity curves were used with an assumed blood volume of 5% to solve for the Logan total distribution volume (DV) and ratio (DVR) using custom MATLAB scripts. Standardized uptake value (SUV) was calculated based on weight and injected dose then used to calculate uptake ratios normalized to the cerebellum (UR).

### Immunohistochemistry

All rats were euthanized with an intraperitoneal injection of pentobarbital (Euthanyl, Bimeda MTC Animal Health Inc) then underwent transcardiac perfusion using 10 mM phosphate-buffered saline (PBS) followed by 4% paraformaldehyde (PFA). Brains were extracted, fixed in 4% PFA for 24 h, then stored at 4 °C in 30% sucrose until they were cryosectioned into 30-µm-thick sections (CryoStar NX50, Thermo Fisher Scientific). Sections were stored in cryoprotectant at + 20 °C until all tissue was available for 3,3′-Diaminobenzidine immunohistochemistry with avidin–biotin complex amplification (ABC Staining Kit, Thermo Fisher Scientific). Primary antibodies were anti-TSPO (1:1000, ab154878, abcam) for TSPO+ cells, anti-iNOS (1:1000, ab15323, abcam) for proinflammatory microglia, anti-OX6 (1:1000, #554926, BD Pharmingen) for MHCII, and anti-GFAP (1:2000, #G3893, Sigma-Aldrich) for activated astrocytes.

The observer was blinded to experimental group during analysis. Images were acquired using a Nikon Eclipse Ni-E microscope and Nikon DS Qi2 color camera on the NIS Elements Imaging software at a consistent lamp voltage and exposure for each antibody. TSPO cells were counted using four 20 × sections at opposite borders of each defined in vivo region except the posterior corpus callosum, which used eight. Weak or diffuse staining was considered negative. GFAP, iNOS, and OX6 within-region % area coverage analysis used large images stitched from 10 × magnification fields. Regions were manually defined using the polygon tool to replicate in vivo delineations of the proximal corpus callosum and forceps minor. The infarct and contralateral regions were also defined for iNOS. Images were binarized with a consistent threshold to get % area coverage within each region (ImageJ 1.52a) [12].

### Statistical analysis

No a priori power analysis was performed as there is no literature data of detecting diffuse WM MHCII microglial activation with TSPO PET in rodent models of subcortical stroke or AD. All analyses of variance (ANOVA) were based on type III sum of squares and considered mixed if there were both between-subject and within-subject variables (SPSS 26). To start, we conducted a two- or three-way ANOVA (depending on whether time was a variable) on PET (between ET1 or genotype, within region and time) and immunohistochemistry (between ET1 and time, within region). Unless stated, higher-order interactions had to meet significance before analyzing simpler effects with lower-order ANOVA and pooled variances if appropriate. Within-subject sphericities were accounted for using Greenhouse–Geisser corrections. Posthoc analyses for repeated timepoint effects were evaluated using Tukey’s honestly significant differences (HSD) unless Shapiro–Wilk normality or Levene’s variance assumptions were violated, in which case the Wilcoxon signed-rank or Mann–Whitney U test were used, depending on whether samples were paired. Correlation and graphing were performed on GraphPad Prism 7. All data is expressed as mean ± standard deviation (SD). Significance was set at alpha = 0.05.

## Results

### The cerebellum is an appropriate pseudoreference region for [^18^F]FEPPA PET

To determine an appropriate pseudoreference region for quantifying [^18^F]FEPPA uptake, [^18^F]FEPPA PET 70–90 min SUVs of the cerebellum and periaqueductal gray were compared (Additional file 1: Online Figs. 1–2 and Online Table 1). The periaqueductal gray was a good candidate for a pseudoreference region because it was usually hypointense relative to the rest of the brain (Additional file 1: Online Fig. 2). The region contralateral to the infarct was also compared in the ET1 investigation. SUV was not significantly affected by genotype, ET1, or interaction between ET1 and timepoints (*P* = ns for two-way and three-way ANOVA). Although it was not significant, it is worthwhile to note that cerebellar SUV appeared elevated at post-stroke day 7. Interestingly, region was a significant factor in both analyses of TgAPP21 vs. wild-type rats (periaqueductal gray = 0.43 ± 0.14; cerebellum = 0.56 ± 0.18; *F*(1,21) = 53.30, *P* < 0*.*0005, two-way ANOVA) and ET1 vs. saline rats (periaqueductal gray = 0.43 ± 0.12; region contralateral to the infarct = 0.49 ± 0.19; cerebellum = 0.60 ± 0.15; *F*(2,12) = 27.47, *P* = 0.001, three-way ANOVA). Although SUV was lowest in the periaqueductal gray, Logan DVR correlated better with DV when the pseudoreference region was the cerebellum instead of the periaqueductal gray or region contralateral to the infarct (Additional file 1: Online Fig. 3). TSPO immunohistochemistry cell count of the day 28 infarct also correlated better with the UR calculated using the cerebellum (R^2^ = 0.63, *P* = 0.0191) instead of the region contralateral to the infarct (R^2^ = 0.47, *P* = ns) or periaqueductal gray (R^2^ = 0.36, *P* = ns) (Additional file 1: Online Fig. 4). Accordingly, cerebellum-based [^18^F]FEPPA UR are reported hereafter.

### [^18^F]FEPPA UR was not elevated in WM of TgAPP21 rats

**Fig. 2:**
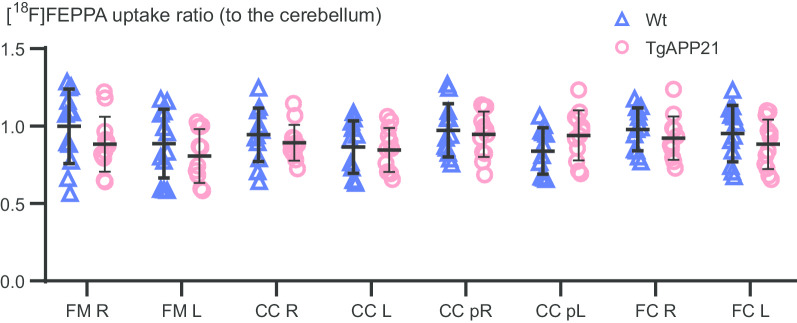
Transgenic amyloid precursor protein rats did not have an elevated [^18^F]FEPPA PET WM uptake ratio, to the cerebellum, relative to wild-type saline rats. Genotype effect was not significant (*P* = ns, two-way ANOVA). CC, corpus callosum; FC, frontal cortex; FM, Forceps Minor; p, posterior. Error = SD

UR in TgAPP21 and wild-type rats were analyzed to investigate [^18^F]FEPPA uptake in WM regions in which our group previously demonstrated MHCII microglial activation [12, 13]. Genotype did not have a significant effect (*P* = ns, two-way ANOVA, Fig. 2). This suggested that [^18^F]FEPPA PET was not detecting the WM MHCII microglia that were previously reported in TgAPP21 rats.

### [^18^F]FEPPA UR was not elevated in remote WM following ET1-induced stroke

**Fig. 3:**
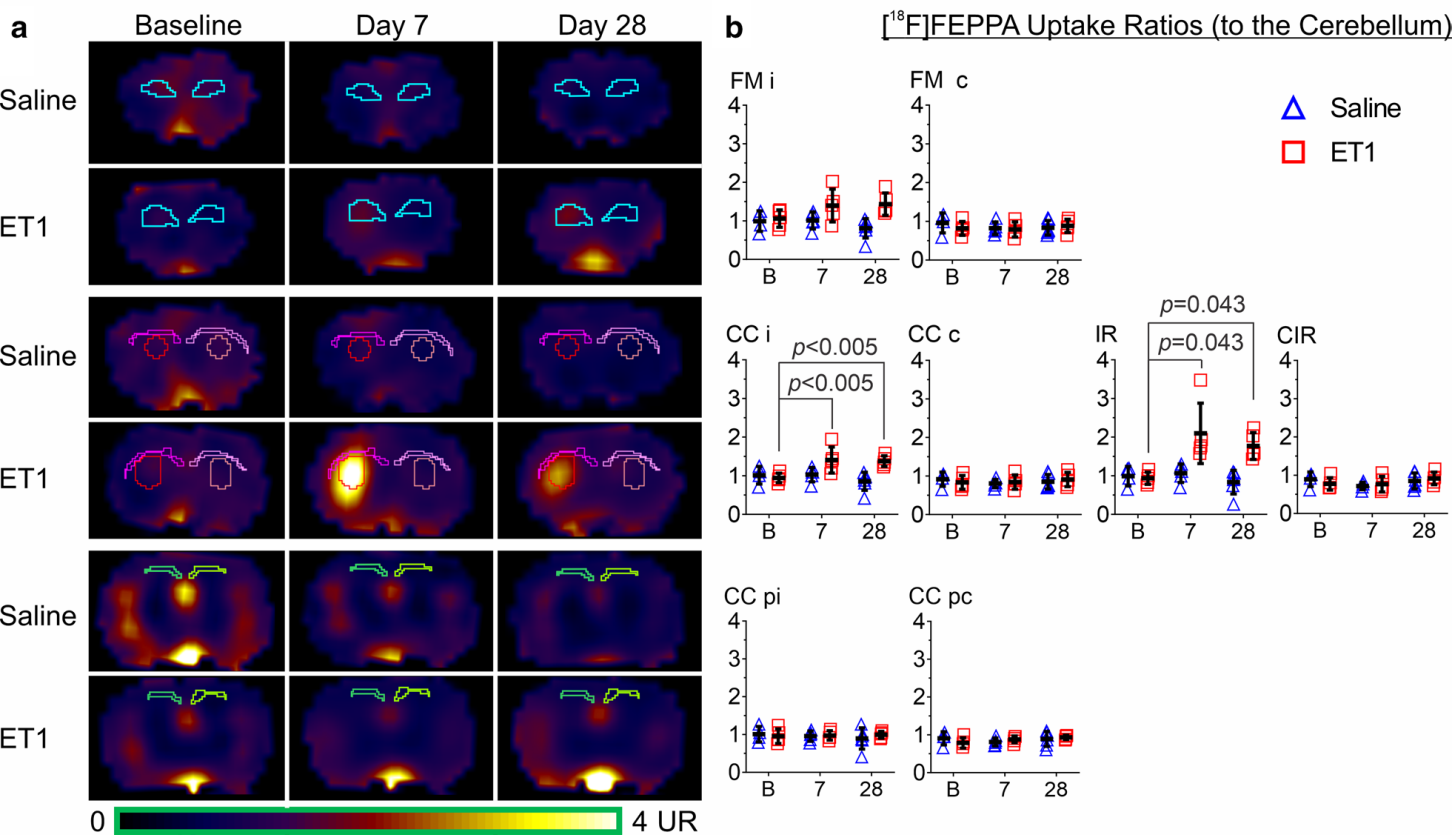
[^18^F]FEPPA UR was not elevated in remote WM following ET1-induced stroke. **a** Maps and **b** values of longitudinal [^18^F]FEPPA PET uptake ratio, relative to the cerebellum, revealed a significant region-ET1-timepoint interaction in saline and ET1 rats [*F*(20,120) = 3.66, *P* = .036, three-way ANOVA]. c, contralateral; CC, corpus callosum; CIR, contralateral to the infarct region; FM, Forceps Minor; i, ipsilateral; IR. infarct region p, posterior. Error = SD

UR at baseline, day 7, and day 28 after injection of ET1 or saline were analyzed to investigate [^18^F]FEPPA uptake in WM with the infarct as a positive control (Fig. 3). An interaction between region, timepoint, and ET1 suggested that UR was increased following ET1-induced stroke only in regions near the infarct [*F*(20,120) = 3.66, *P* = 0.036, three-way ANOVA]. In the infarct ROI of the ET1 group, UR significantly increased from baseline (0.94 ± 0.16) to day 7 (2.10 ± 0.78; *P* = 0.043, Wilcoxon signed-rank) and day 28 (1.77 ± 0.35; *P* = 0.043, Wilcoxon signed-rank). Similarly, proximal WM (ipsilateral corpus callosum) UR of the ET1 group significantly increased from baseline (0.94 ± 0.12) to day 7 (1.42 ± 0.34; *P* < 0.005, Tukey’s HSD) and day 28 (1.38 ± 0.14; *P* < 0.005, Tukey’s HSD). UR was not significantly affected in remote WM.

### TSPO was not expressed in remote WM following ET1-induced stroke

**Fig. 4:**
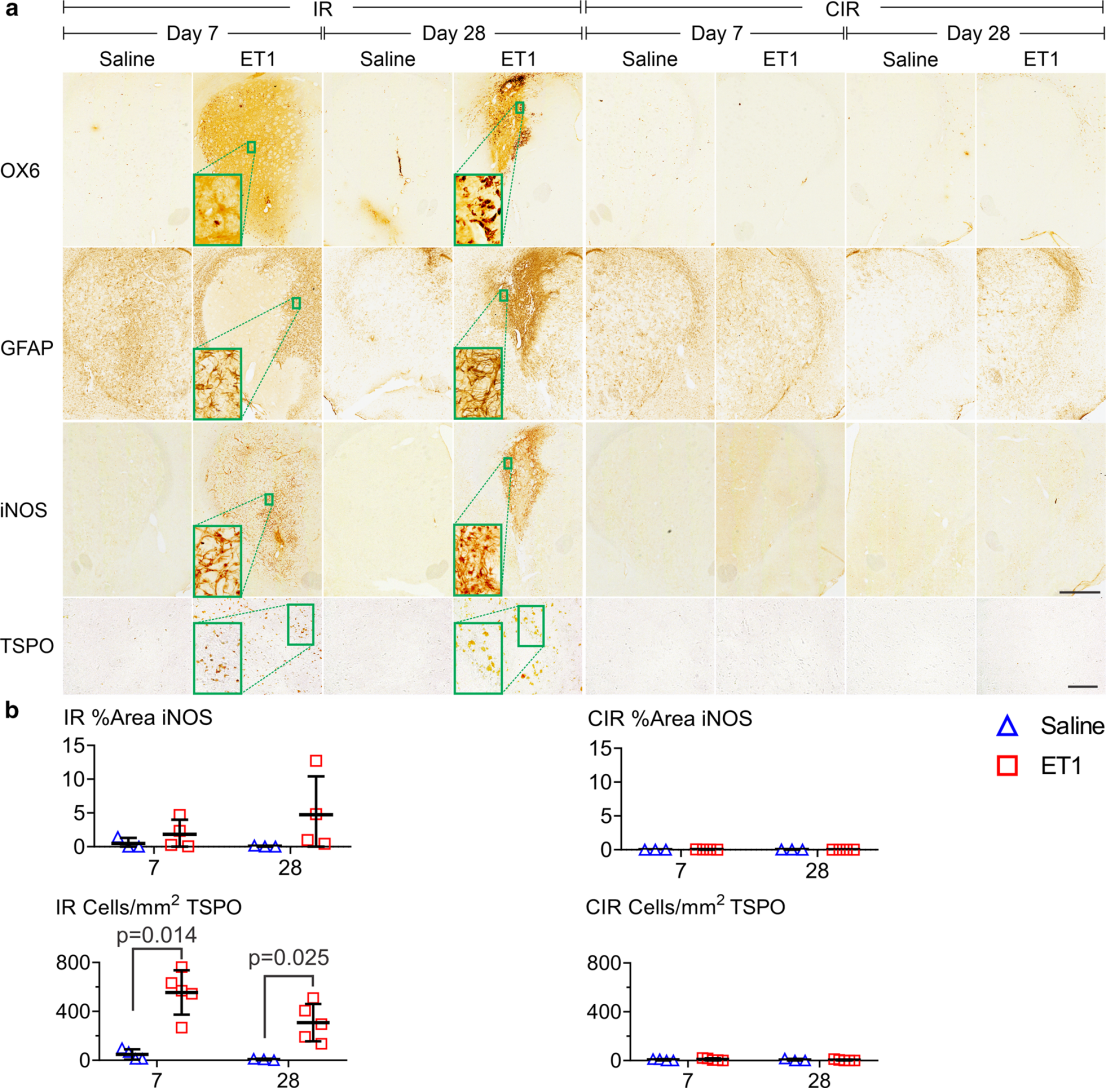
Immunohistochemistry showed that TSPO, but not iNOS, was elevated in the infarct site of ET1 rats at days 7 and 28 post-stroke. **a** Representative images of OX6 (MHCII), GFAP, iNOS, and TSPO in the infarct region (IR) and contralateral to the IR (CIR) post-stroke at day 7 and day 28 in saline and ET1 rats. **b** Quantification of cell count for TSPO and % area coverage for iNOS. Bar indicates 1 mm in large images and 100 µm in TSPO images. Error = SD

**Fig. 5:**
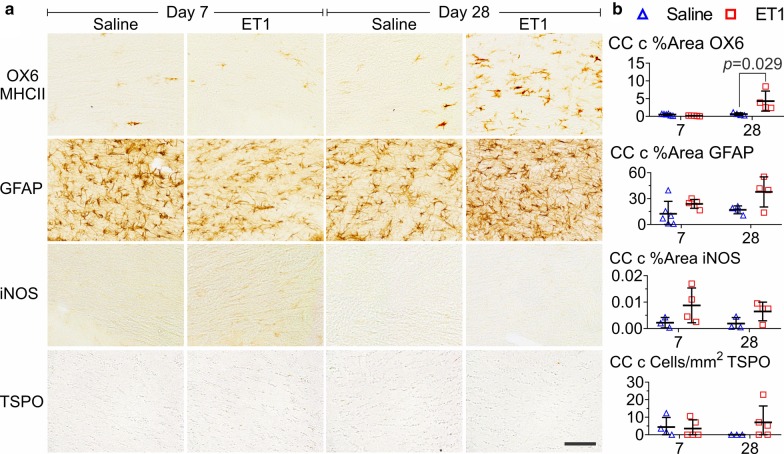
WM immunohistochemistry in the contralateral corpus callosum (CC c) showing that at 28 days post-stroke, ET1 rats only showed an elevation of OX6 (MHCII); not TSPO, iNOS, and GFAP. **a** Representative images and **b** quantification of cell count for TSPO and % area coverage for other markers. Bar indicates 100 µm. Error = SD

TSPO immunohistochemistry-based cell counting was performed to confirm [^18^F]FEPPA PET findings in stroke. An interaction between region and ET1 suggested that TSPO immunohistochemistry concurred with [^18^F]FEPPA on the finding that ET1 increased TSPO only in regions near the infarct [*F*(9,117) = 27.04, *P* < 0.0005, three-way ANOVA, Fig. 4]. In the infarct, ET1 significantly increased TSPO cell count at day 7 (cells/mm^2^: saline = 48 ± 43; ET1 = 555 ± 181; *P* = 0.014, Mann Whitney U test) and day 28 (cells/mm^2^: saline = 6 ± 6; ET1 = 307 ± 153; *P* = 0.025, Mann Whitney U test). Proximal WM (ipsilateral corpus callosum) TSPO cell count agreed with [^18^F]FEPPA PET in regards to a day 7 elevation (113 ± 93 cells/mm^2^) but disagreed by showing no day 28 elevation (5 ± 7 cells/mm^2^) (Additional file 1: Online Fig. 5). Remote WM TSPO cell counts were low and unaffected by stroke (Fig. 5 and Additional file 1: Online Fig. 5). Recognizing that spillover of PET signal from the infarct into the ipsilateral corpus callosum might indicate an increased uptake in [^18^F]FEPPA PET when there is no TSPO overexpression, we used TSPO immunohistochemistry for further comparison of TSPO with GFAP, iNOS, and MHCII.

### GFAP and iNOS expression was not changed in WM following ET1-induced stroke

GFAP and iNOS immunohistochemistry were performed to further investigate changes in astrocytes and microglia as these markers are often compared with TSPO [21, 22]. GFAP signal showed a day 7 border around the infarct and a day 28 scar in ET1 rats, but there were no significant effects in WM (*P* = ns, three-way ANOVA, Fig. 4). iNOS signal showed sensitivity to ET1 only in the infarct (% area: day 7 = 1.84 ± 2.16; day 28 = 4.74 ± 5.65; *P* = ns), without significance, and was not elevated in WM (*P* = ns, three-way ANOVA) (Figs. 4 and 5). Overall, GFAP and iNOS concurred with TSPO immunohistochemistry, with no significant changes in remote WM following ET1-induced stroke.

### MHCII positive activated microglia were chronically expressed in remote WM following ET1-induced stroke

Immunohistochemistry of the MHCII rat antigen OX6 was used to confirm previous findings by our group of an ET1-induced increase of remote WM MHCII positive microglia activation (Fig. 5) [14, 15]. WM OX6 signal was significantly affected by an interaction between timepoint and ET1 [*F*(1,15) = 9.73, *P* = 0.007, three-way ANOVA]. Although there were no significant interactions with region, we separately analyzed the contralateral corpus callosum at day 28 and found a significant effect of ET1 (% area saline = 0.62 ± 0.38, ET1 = 4.30 ± 2.83; *P* = 0.029, Mann Whitney U test). This indicated that by day 28 post-stroke, MHCII was the only marker to show sensitivity to ET1 in WM.

## Discussion

The literature about the non-specificity of TSPO to activated microglia is growing, but there are few reports showing that TSPO does not colocalize with all activated microglia or is not as sensitive as MHCII [23–25]. We found that [^18^F]FEPPA PET did not capture the expected increase of diffuse, chronically activated MHCII microglia in WM of TgAPP21 rats. Additionally, in an ET1-induced subcortical stroke model, MHCII microglia in remote WM were not detectable by [^18^F]FEPPA PET or TSPO immunohistochemistry.

The cerebellum was found to be an appropriate pseudoreference region. Other rodent AD studies have shown that the cerebellum is an acceptable pseudoreference region by demonstrating a lack of group effect on SUV [26]. When multiple pseudoreference regions are assessed in stroke, if the group effect is null for multiple regions, preference is sometimes given to the region with the lowest uptake [27]. Although SUV was overall lower in the periaqueductal gray and region contralateral to the infarct, and cerebellar SUV post-stroke at day 7 was non-significantly elevated, we found that calculating UR using the cerebellum provided better correlations with TSPO immunohistochemistry and between DV and DVR. Using the cerebellum also ensured that our WM ROIs are sufficiently distant, thereby minimizing bias caused by partial volume effects. All of this suggests that the cerebellum is a practical pseudoreference for rodent TSPO PET studies of stroke and AD, but others should continue to investigate and validate their choice.

Although an increase in uptake of TSPO tracers in the infarct has been well documented along with the cellular source of TSPO using other microglial markers such as Cd11b and Iba1, to our knowledge, rodent studies have only investigated cortical strokes as opposed to subcortical strokes [21–24, 27]. In our study, we found that ET1-induced subcortical stroke infarcts have increased TSPO uptake at days 7 and 28 post-stroke. The proportion of TSPO signal from astrocytes cannot be deduced without multilabel immunofluorescence, but the formation of a GFAP-positive astrocyte scar between days 7 and 28 suggests a change in astrocyte function that might explain why TSPO expression increases in astrocytes with time after a stroke [21, 23, 24]. Similarly, microglial functions seem to change as proinflammatory markers differ longitudinally; after day 7, some proinflammatory markers decrease while others, including iNOS, increase [28]. Our study similarly demonstrated that after day 7, iNOS increases while TSPO decreases, albeit insignificant partly due to high variability in rodent post-stroke iNOS expression *(28)*. Moreover, studies of cortical strokes have used a variety of multilabel immunofluorescence combinations to investigate the cellular origin of TSPO in stroke. For these reasons, TSPO PET may not provide unequivocal, longitudinal measurements for guiding subject-specific infarct-targeted anti-inflammatory therapy after stroke in rodent models.

Our findings suggest that TSPO does not capture diffuse WM inflammation in the TgAPP21 model or remote WM inflammation in the ET1-induced subcortical stroke model. WM TSPO studies in rodents have been restricted to mouse models of multiple sclerosis, which have focally elevated TSPO in active demyelinating lesions [30]. In the current study, prodromal AD TgAPP21 rats did not take up more [^18^F]FEPPA according to PET in WM, despite having previously demonstrated diffuse WM MHCII activated microglia [12]. Similarly, in ET1-induced subcortical stroke, WM TSPO expression was only increased in regions with focal inflammation and not in regions with diffuse inflammation. To our knowledge, TSPO overexpression in rodent WM without a nearby lesion has yet to be demonstrated.

TSPO mismatch with MHCII might be dependent on whether the pathology is associated with antigens that would induce the antigen-presenting function of MHCII in microglia. When antigens are not expected to induce pathology, TSPO is overexpressed without MHCII, as was shown in a rat model of alcohol-induced neurodegeneration [31]. Conversely, when lesions are present with antigenic alpha-synuclein, as in cases of human multiple system atrophy, post-mortem tissue had a 6-fold increase in MHCII and 2-fold increase in TSPO relative to controls [25]. Our work in rats demonstrates that although TSPO detected neuroinflammation near the ET1-induced infarct, only MHCII was able to detect the WM microglial activation that occurred late post-stroke or in response to overexpression of pathogenic APP. Although this makes MHCII a desirable target for PET, its many genetic variants may pose a challenge. Additionally, MHCII may be expressed by the other antigen-presenting cells such as dendritic cells and B cells, but further investigation is needed as MHCII expression in the brain has currently only been attributed to microglia and neural progenitor cells to our knowledge [9, 32].

This study has two main limitations. First, rat WM is small and susceptible to limits in PET resolution and sensitivity. A higher resolution (50–100 μm) and improved binding context may be achieved using autoradiography, which does this by allowing sections to be extracted from the body of a subject and (i) placed closer to high-resolution detectors than what is used in PET and (ii) the radiotracer does not need to pass a blood compartment and can be exposed to toxically high non-radioactive ligand amounts that can outcompete low-affinity nonspecific binding [33]. Although autoradiography could greatly help evaluate the binding specificity and estimate the whereabouts of [^18^F]FEPPA uptake better than [^18^F]FEPPA PET, we were overall interested in the expression of TSPO itself. Accordingly, we opted for the higher-resolution (< 1 μm) TSPO immunohistochemistry in rats injected with ET1 or saline to validate that TSPO is not sensitive to WM MHCII activated microglia. As immunohistochemistry was not performed for the TgAPP21 rats, it is possible that they had elevated TSPO expression in the WM, but unlikely given that their PET [^18^F]FEPPA UR was lower, although insignificantly, than that of Wt rats. Second, this study used only males to minimize variables as females differ in their TSPO expression and neuroinflammatory response [34]. Whether males and females differ in their TSPO expression at an infarct or in WM should be further investigated.

In conclusion, we found that an ET1-induced subcortical stroke increases TSPO expression. More importantly, WM microglia only expressed TSPO near an ET1-induced lesion, thereby revealing that TSPO was insensitive to MHCII activated microglia in remote WM and in WM of prodromal AD rats without a lesion. An MHCII radiotracer would enable longitudinal imaging of chronically activated MHCII microglia in neurodegenerative diseases.

## Supplementary information

**Additional file 1.: Online Fig. 1** SUV was significantly different between the pseudoreference regions, cerebellum and PAG, in wild-type and TgAPP21 [*F*(1,21) = 53.30,* P* < .0005, two-way ANOVA]. Whole brain SUV is also shown. CIR: contralateral to the infarct region. PAG: periaqueductal gray. Error = SD. **Online Fig. 2** Wild-type saline and ET1 rat pseudoreference regions at baseline, day 7, and day 28 after an injection in the right dorsal striatum. (A) SUV maps overlaid on T2-weighted MRI. (B) SUV showed a significant effect of region [*F*(1,21) = 53.30,* P* < .0005, two-way ANOVA]. CIR, contralateral to the infarct region; PAG, periaqueductal gray. Error = SD. **Online Table 1** SUV (mean ± SD) in the pseudoreference regions of wild-type saline and ET1 rats at baseline, day 7, and day 28 after an injection in the right dorsal striatum. SUV was not significantly affected by genotype, ET1, or interaction between ET1 and timepoints (*P* = ns for two-way and three-way ANOVA). Region was a significant factor (*F*(2,12) = 27.47,* P* = 0.001, three-way ANOVA). **Online Fig. 3** Correlations of distribution volume with distribution volume ratio to candidate pseudoreference regions. Each point represents one region in one Wt rat at the 28 day post-stroke timepoint. The coefficients of determination are provided in the graph. CIR, contralateral to the infarct region; PAG, periaqueductal gray. **Online Fig. 4** Correlations of infarct TSPO immunohistochemistry to uptake ratios calculated using candidate pseudoreference regions. Regression coefficients are provided in the graph. CIR, contralateral to the infarct region; PAG, periaqueductal gray; IR, Infarct region. **Online Fig. 5** TSPO Immunohistochemistry of WM at day 7 and day 28 in saline and ET1 rats. (A) Representative images and (B) quantification of cell count for TSPO. FM, Forceps Minor; CC, corpus collosum; i, ipsilateral; c, contralateral; p, posterior. Bar indicates 100 µm. Error = SD.

## Data Availability

All data generated or analysed during this study are included in this published article and its supplementary information files.

## Abbreviations

WM: White matter
AD: Alzheimer’s disease
PET: Positron emission tomography
TSPO: Translocator protein
MHCII: Major histocompatibility complex class II
TgAPP21: Transgenic Fischer 344 rat that overexpresses human Swedish/Indiana-mutated amyloid precursor protein
[^18^F]FEPPA: [^18^F]-N-(2-(2-fluoroethoxy)benzyl)-n-(4-phenoxypyridin-3-yl)acetamide
ET1: Endothelin-1
FSE: Fast spin echo
FOV: Field of view
TR: Repetition time
CT: Computed tomography
OSEM3D/FMAP: 3D ordered-subset expectation-maximization followed by fast maximum a posteriori
ROI: Region of interest
DV: Total distribution volume
DVR: DV ratio
SUV: Standardized uptake value
UR: Uptake ratio normalized to the cerebellum
