# Supplementary material for: TSPO PET detects acute neuroinflammation but not diffuse chronically activated MHCII microglia in the rat
Source: EJNMMI Res. 2020 Sep 29;10:113. doi: 10.1186/s13550-020-00699-x (PMC7524910; doi:10.1186/s13550-020-00699-x)
Supplement: Supplementary file 1 — Additional file 1.: Online Fig. 1 SUV was significantly different between the pseudoreference regions, cerebellum and PAG, in wild-type and TgAPP21 [F(1,21) = 53.30, P < .0005, two-way ANOVA]. Whole brain SUV is also shown. CIR: contralateral to the infarct region. PAG: periaqueductal gray. Error = SD. Online Fig. 2 Wild-type saline and ET1 rat pseudoreference regions at baseline, day 7, and day 28 after an injection in the right dorsal striatum. (A) SUV maps overlaid on T2-weighted MRI. (B) SUV showed a significant effect of region [F(1,21) = 53.30, P < .0005, two-way ANOVA]. CIR, contralateral to the infarct region; PAG, periaqueductal gray. Error = SD. Online Table 1 SUV (mean ± SD) in the pseudoreference regions of wild-type saline and ET1 rats at baseline, day 7, and day 28 after an injection in the right dorsal striatum. SUV was not significantly affected by genotype, ET1, or interaction between ET1 and timepoints (P = ns for two-way and three-way ANOVA). Region was a significant factor (F(2,12) = 27.47, P = 0.001, three-way ANOVA). Online Fig. 3 Correlations of distribution volume with distribution volume ratio to candidate pseudoreference regions. Each point represents one region in one Wt rat at the 28 day post-stroke timepoint. The coefficients of determination are provided in the graph. CIR, contralateral to the infarct region; PAG, periaqueductal gray. Online Fig. 4 Correlations of infarct TSPO immunohistochemistry to uptake ratios calculated using candidate pseudoreference regions. Regression coefficients are provided in the graph. CIR, contralateral to the infarct region; PAG, periaqueductal gray; IR, Infarct region. Online Fig. 5 TSPO Immunohistochemistry of WM at day 7 and day 28 in saline and ET1 rats. (A) Representative images and (B) quantification of cell count for TSPO. FM, Forceps Minor; CC, corpus collosum; i, ipsilateral; c, contralateral; p, posterior. Bar indicates 100 µm. Error = SD. [file 13550_2020_699_MOESM1_ESM.docx]

**TSPO PET Detects Acute Neuroinflammation but not Diffuse Chronically Activated MHCII Microglia in the Rat**

Nassir U. Al-Khishman^1,5^, Qi Qi^1,5^, Austyn D. Roseborough^2^, Alexander Levit^2^, Brian L. Allman^2^, Udunna C. Anazodo^1,4^, Matthew S. Fox^3,5^, Shawn N. Whitehead^2^* and Jonathan D. Thiessen^1,5^*

*indicates both authors contributed equally.

**Affiliations:**

^1^ Department of Medical Biophysics, Schulich School of Medicine & Dentistry, Western University, London, Ontario, Canada

^2^ Department of Anatomy & Cell Biology, Schulich School of Medicine & Dentistry, Western University, London, Ontario, Canada

^3^ Department of Physics and Astronomy, Western University, London, Ontario, Canada N6A 3K7

^4^ Research Centre for Studies in Aging, McGill University, Montreal, Quebec, Canada H4H 1R3

^5^ Lawson Health Research Institute, London, Ontario, Canada N6A 4V2

**Corresponding Author:** Jonathan D. Thiessen, B5-003a, 268 Grosvenor St, P.O. Box 5777, Stn. B London, Ontario, Canada N6A 4V2. V: (519)-646-6100 X 64181. F: (519)-646-6205 ([jthiessen@lawsonimaging.ca](mailto:jthiessen@lawsonimaging.ca))


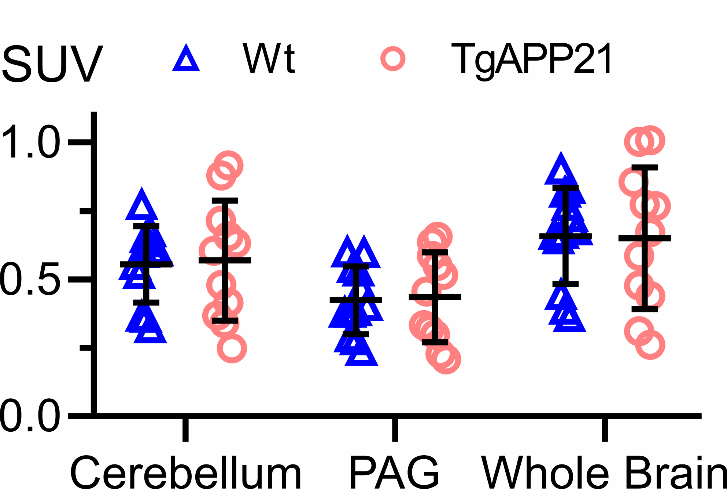
**SUPPLEMENTARY DATA**

**Online Fig 1** SUV was significantly different between the pseudoreference regions, cerebellum and PAG, in wild-type and TgAPP21 [F(1,21)=53.30, P<.0005, two-way ANOVA]. Whole brain SUV is also shown. CIR: contralateral to the infarct region. PAG: periaqueductal gray. Error = SD

*
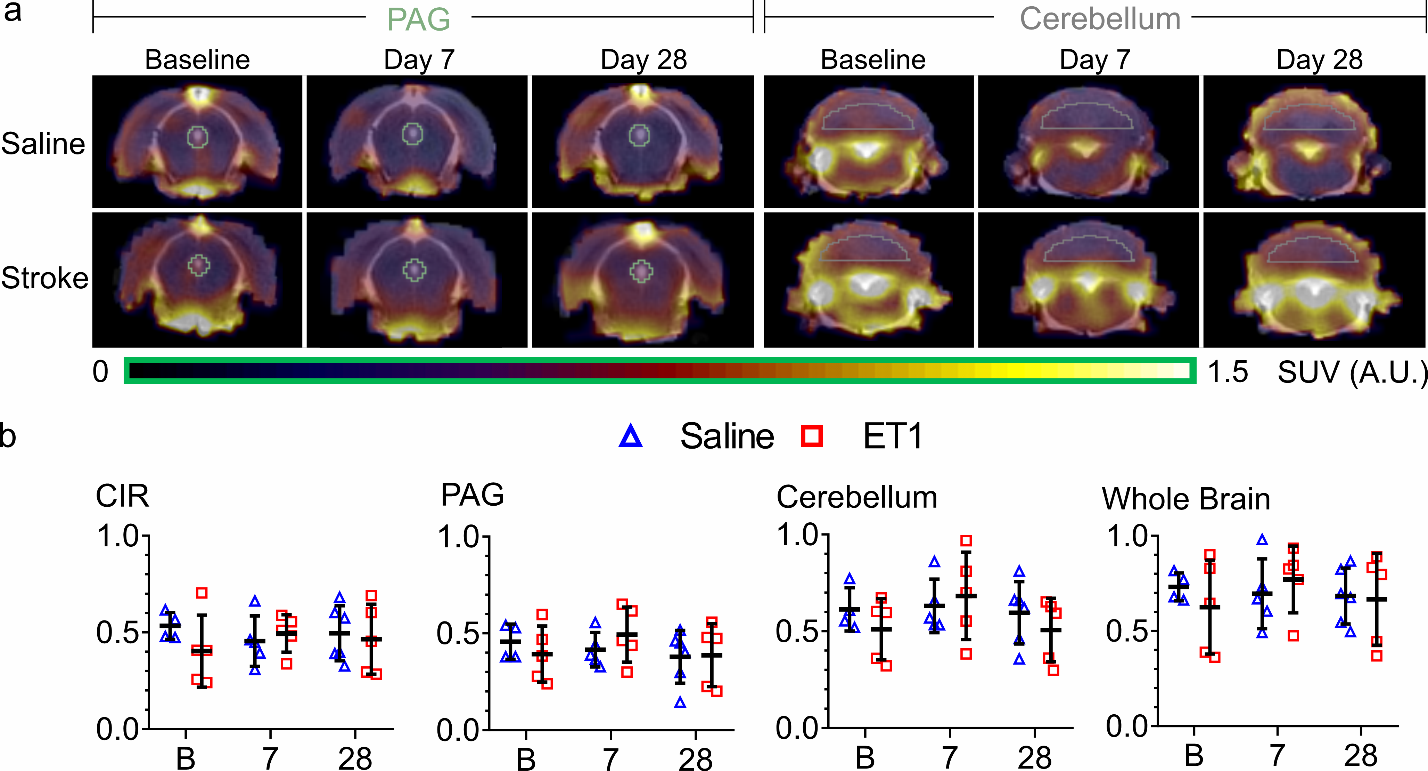
*

**Online Fig 2** Wild-type saline and ET1 rat pseudoreference regions at baseline, day 7, and day 28 after an injection in the right dorsal striatum. (A) SUV maps overlaid on T_2_-weighted MRI. (B) SUV showed a significant effect of region [F(1,21)=53.30, P<.0005, two-way ANOVA]. CIR, contralateral to the infarct region; PAG, periaqueductal gray. Error = SD

**Online Table 1** SUV (mean ± SD) in the pseudoreference regions of wild-type saline and ET1 rats at baseline, day 7, and day 28 after an injection in the right dorsal striatum. SUV was not significantly affected by genotype, ET1, or interaction between ET1 and timepoints (P = ns for two-way and three-way ANOVA). Region was a significant factor (*F*(2,12) = 27.47, *P =* 0.001, three-way ANOVA).

| Group | CIR † | |  | PAG† | |  | Cerebellum | |  | Whole Brain | |
| --- | --- | --- | --- | --- | --- | --- | --- | --- | --- | --- | --- |
|  | Saline | ET1 |  | Saline | ET1 |  | Saline | ET1 |  | Saline | ET1 |
| Baseline | 0. 53 ± 0.07 | 0. 40 ± 0.19 |  | 0. 46 ± 0.09 | 0. 39 ± 0.15 |  | 0. 61 ± 0.11 | 0. 51 ± 0.16 |  | 0. 73 ± 0.07 | 0. 63 ± 0.35 |
| Day 7 | 0. 46 ± 0.13 | 0. 49 ± 0.10 |  | 0. 42 ± 0.09 | 0. 49 ± 0.14 |  | 0. 63 ± 0.14 | 0. 68 ± 0.23 |  | 0. 70 ± 0.18 | 0. 77 ± 0.18 |
| Day 28 | 0. 50 ± 0.14 | 0. 47 ± 0.18 |  | 0. 38 ± 0.14 | 0. 39 ± 0.16 |  | 0. 60 ± 0.16 | 0. 51 ± 0.16 |  | 0. 68 ± 0.15 | 0. 67 ± 0.24 |

† CIR: Contralateral to the infarct region
† PAG: Periaqueductal gray


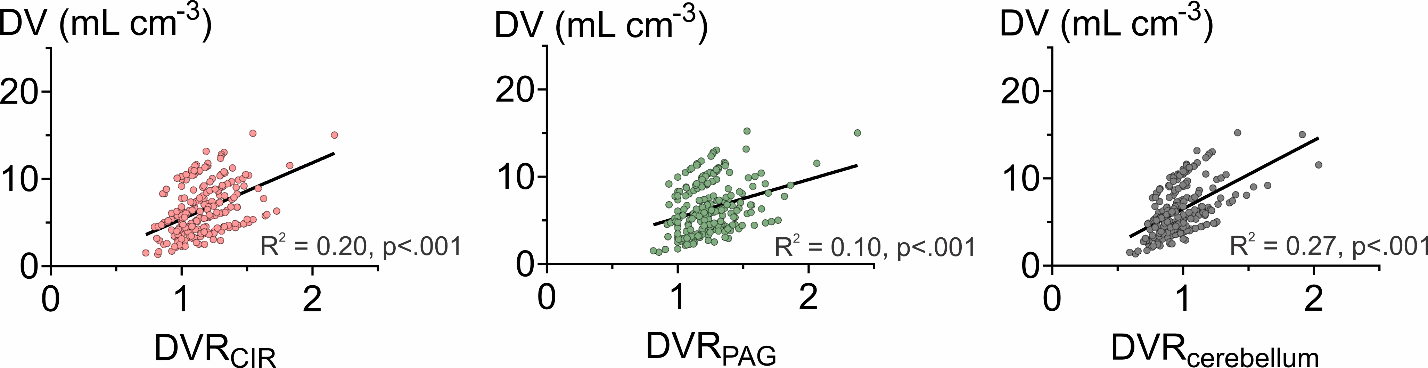


**Online Fig 3** Correlations of distribution volume with distribution volume ratio to candidate pseudoreference regions. Each point represents one region in one Wt rat at the 28 day post-stroke timepoint. The coefficients of determination are provided in the graph. CIR, contralateral to the infarct region; PAG, periaqueductal gray


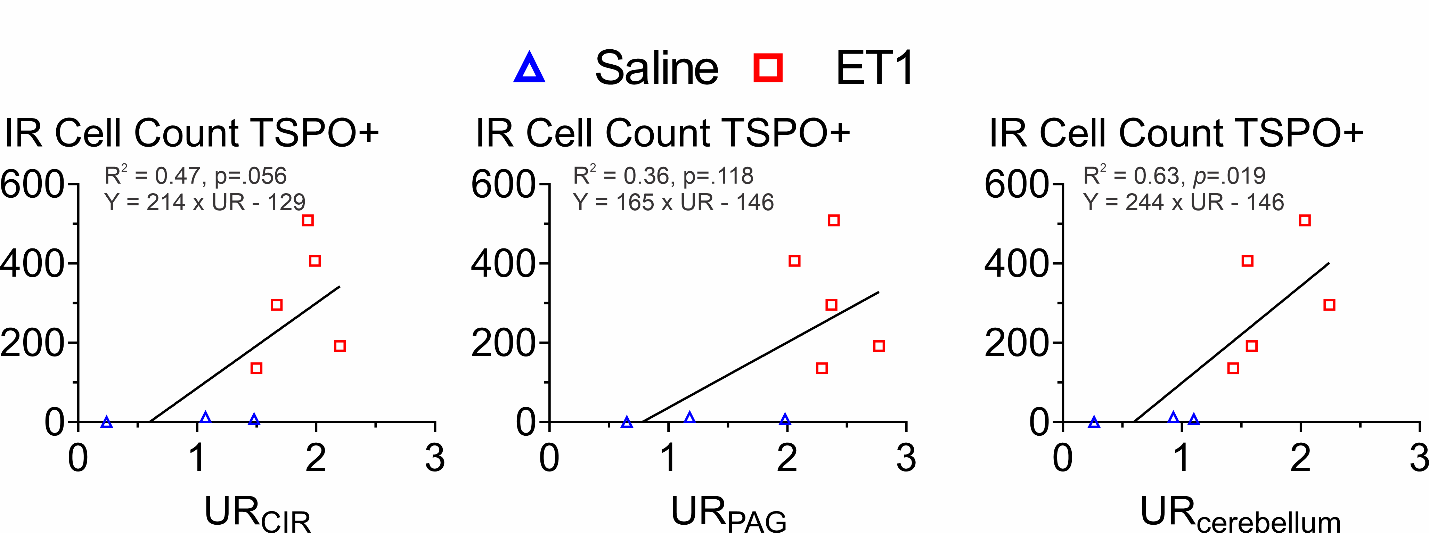


**Online Fig 4** Correlations of infarct TSPO immunohistochemistry to uptake ratios calculated using candidate pseudoreference regions. Regression coefficients are provided in the graph. CIR, contralateral to the infarct region; PAG, periaqueductal gray; IR, Infarct region


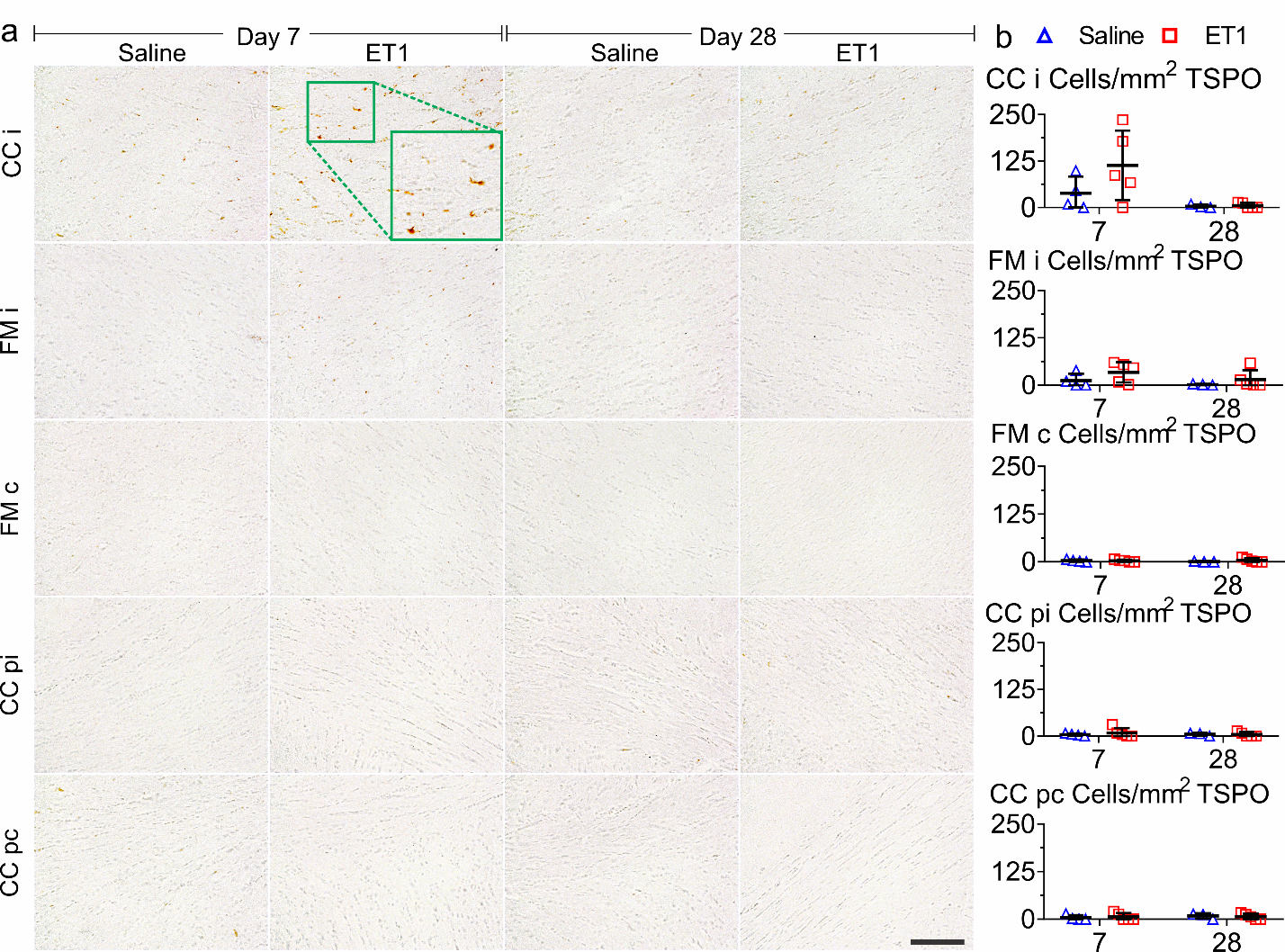


**Online Fig 5** TSPO Immunohistochemistry of WM at day 7 and day 28 in saline and ET1 rats. (A) Representative images and (B) quantification of cell count for TSPO. FM, Forceps Minor; CC, corpus collosum; i, ipsilateral; c, contralateral; p, posterior. Bar indicates 100 µm. Error = SD
